# RETRACTION: Melatonin Alleviated Potassium Dichromate‐Induced Oxidative Stress and Reprotoxicity in Male Rats

**DOI:** 10.1155/bmri/9758290

**Published:** 2026-04-17

**Authors:** BioMed Research International

RETRACTION: S. A. E. Bashandy, H. Ebaid, J. Al‐Tamimi, O. A‐H. Ahmed‐Farid, E. A. Omara, I. M. Alhazza, “Melatonin Alleviated Potassium Dichromate‐Induced Oxidative Stress and Reprotoxicity in Male Rats,” *BioMed Research International*, 2021, 3565360, https://doi.org/10.1155/2021/3565360

The above article, published online on 16 June 2021 in Wiley Online Library (wileyonlinelibrary.com), has been retracted by agreement between the journal handling Editor, Valeria Pasciu, and John Wiley & Sons Ltd. The retraction has been agreed due to inconsistencies identified in Figures 3 and 4, initially identified on PubPeer [1]. On further investigation, additional concerns with the figures were identified. Specifically:

• In Figure 3, there are unexpected repeated elements between panels A and D.
• In Figure 4, there are unexpected repeated elements between panels A and E.
• In Figure 4, there are unexpected repeated elements within panel A.
• In Figure 4, there are unexpected repeated elements within panel E.

After assessment of the concerns and author’s response by the editorial board, the results and conclusions are deemed unreliable and the article is therefore retracted.

The authors disagree with the retraction.

## References

[1] belfragei A. , Melatonin Alleviated Potassium Dichromate-Induced Oxidative Stress and Reprotoxicity in Male Rats, PubPeer. (2024) https://pubpeer.com/publications/62CB3B941F42F782695C9B8C4007D8. 10.1155/2021/3565360 PMC8221856 34222468

